# Dynamic virtual fixture on the Euclidean group for admittance-type manipulator in deforming environments

**DOI:** 10.1186/1475-925X-13-51

**Published:** 2014-04-27

**Authors:** Dongwen Zhang, Qingsong Zhu, Jing Xiong, Lei Wang

**Affiliations:** 1 Shenzhen Key Laboratory for Lowcost Healthcare, Key Lab for Health Informatics, Shenzhen Institutes of Advanced Technology, Chinese Academy of Sciences, Xueyuan Avenue 1068, Shenzhen 518055, China; 2 University of Chinese Academy of Sciences, No.19A Yuquan Road, Beijing 100049, China

## Abstract

**Background:**

In a deforming anatomic environment, the motion of an instrument suffers from complex geometrical and dynamic constraints, robot assisted minimally invasive surgery therefore requires more sophisticated skills for surgeons. This paper proposes a novel dynamic virtual fixture (DVF) to enhance the surgical operation accuracy of admittance-type medical robotics in the deforming environment.

**Methods:**

A framework for DVF on the Euclidean Group *SE*(3) is presented, which unites rotation and translation in a compact form. First, we constructed the holonomic/non-holonomic constraints, and then searched for the corresponded reference to make a distinction between preferred and non-preferred directions. Second, different control strategies are employed to deal with the tasks along the distinguished directions. The desired spatial compliance matrix is synthesized from an allowable motion screw set to filter out the task unrelated components from manual input, the operator has complete control over the preferred directions; while the relative motion between the surgical instrument and the anatomy structures is actively tracked and cancelled, the deviation relative to the reference is compensated jointly by the operator and DVF controllers. The operator, haptic device, admittance-type proxy and virtual deforming environment are involved in a hardware-in-the-loop experiment, human-robot cooperation with the assistance of DVF controller is carried out on a deforming sphere to simulate beating heart surgery, performance of the proposed DVF on admittance-type proxy is evaluated, and both human factors and control parameters are analyzed.

**Results:**

The DVF can improve the dynamic properties of human-robot cooperation in a low-frequency (0 ~ 40 rad/sec) deforming environment, and maintain synergy of orientation and translation during the operation. Statistical analysis reveals that the operator has intuitive control over the preferred directions, human and the DVF controller jointly control the motion along the non-preferred directions, the target deformation is tracked actively.

**Conclusions:**

The proposed DVF for an admittance-type manipulator is capable of assisting the operator to deal with skilled operations in a deforming environment.

## Background

In a deforming anatomic environment, minimally invasive surgery (MIS) therefore requires more sophisticated skills for the surgeons, the motion of an instrument suffers from complex geometrical and dynamic constraints. One approach for enhancing the accuracy and safety of human operation is to use robot controller to regulate the instrument motion under either hands-on or teleoperation control. These are referred to as virtual fixtures (VF), which is kind of task-dependent and computer-generated mechanisms to limit movement into restricted regions [1-3] or influence movement along desired paths [4-8]. Extension of VF into deforming environment also refers to dynamic virtual fixture [9], where the geometric constraint moves continuously, as a result of changes in the physical environment or task being undertaken [10,11].

An important case of VFs for surgical application is guidance VF, Bettini et al. [5,6] used vision information to assist VF construction, and they discussed the application in vitreoretinal surgery. Marayong et al. [7] demonstrated a geometrical constraint with varying compliance, which is described for the general spatial case. Compliant control is significantly necessary to overcome the uncertainties associated with registration errors, variations in anatomy [12]. Li et al. [13] presented an anatomy generated VF for sinus surgery, which employed a constrained optimization framework to incorporate task goals, anatomy-based constraints, forbidden zones, etc. Castillo-Cruces et al. [14] presented an admittance controller with autonomous error compensation, a clear distribution of responsibilities between surgeon and robotic system is preserved. Becker et al. [15] derived a virtual fixture framework for active handheld micromanipulators, the forces were replaced with an actuated tool tip that is permitted by the high-bandwidth position measurements. These work all used admittance controller to implement VFs, the active robots comply with the manual force that applied on the end-effector or telemanipulated joystick. However, such first-order admittance controller with manual/autonomous error compensation is inadequate to adapt to dynamic environment. Bebek et al. proposed an intelligent control algorithm for robotic-assisted beating heart surgery, the robotic tools actively cancel the relative motion between the surgical instruments and the point of interest on the beating heart [16]. However, motions along the preferred directions are not regulated.

Traditional VFs deal with rotation and translation separately in ℝ^3^ space, the interconnection between them is not involved in VF design. Synergy of orientation and position is important for MIS, it requires that the orientation should be ready before the position is reached. Otherwise, a delay is needed to instrument motion when the orientation and position are out of synchronous, it could be awful for real-time operation, collisions could probably appears in the inner anatomic operation space. Bullo and Murray [17] proposed a generalized proportional derivative (PD) laws on Euclidean Group *SE*(3), which used logarithmic feedback control and dealt with rotation and translation simultaneously with a compact form. Besides, the geometric properties of Lie group and Lie algebra facilitate spatial compliance and stiffness matrix synthesis and decomposition. Shuguang Huang [18,19] examined the structure of spatial stiffness by evaluating the eigenscrew featured rank-1 primitives stiffness matrices.

In our previous works, we studied the spatial compliance/stiffness matrices synthesis for admittance and impedance controlled devices respectively [20].

The proposed VFs could not effectively eliminate the tracking error drifts over the deforming frequency growth, thus we hope to improve the dynamic performance of the VF for admittance controlled device. In this paper, we study the application of VF in deforming environment, and propose a novel framework of DVF for admittance-type device on *SE*(3). We construct the holonomic constraint/non-holonomic constraints and then search for the corresponded reference tasks to make a distinction between preferred and non-preferred directions. Different control strategies are employed to deal with the tasks along the distinguished directions. The paper is organized as follows: the first section outlines the mathematical preliminaries of Lie group and Lie algebra for robot control, spatial compliance matrix synthesis and DVF on *SE*(3) for admittance-type device are proposed in Methods the last two sections describe a hardware-in-the-loop experiment and discussions.

## Methods

### Mathematical preliminaries

The configuration space of a robotic system is defined on the special Euclidean group *SE*(3) and its subgroups, position and orientation of the end-effector correspond to a element of matrix Lie group. The *Lie algebra* of *SE*(3) is denoted as *se*(3). We express a group element g = (**R**, *p*) ∊ *SE*(3) and velocity $V=\left(\hat{\omega },v\right)\in \mathrm{se}\left(3\right)$ using homogeneous coordinates as

(1) $$g=\left[\begin{array}{cc} R & p\\ 0 & 1 \end{array}\right],\;V=\left[\begin{array}{cc} \hat{\omega } & v\\ 0 & 0 \end{array}\right]$$

the operator $\hat{*}:{\mathbb{R}}^{3}\to \mathrm{so}\left(3\right)$ is a cross-product matrix that transforms cross-product operation into matrix multiplication, so that $\hat{x}y=x\times y$ for all ***x***, ***y*** ∈ **R**^3^. Elements of *se*(3) can also be represented as vector pair (***ω***, ***v***), ***ω***, ***v*** ∈ ℝ^3^.

For all g ∊ *SE*(3) and all **X**, **Y** ∊ *se*(3), the *Adjoint map*${\mathrm{Ad}}_{g}:\mathrm{se}\left(3\right)\to \mathrm{se}\left(3\right)$ is a coordinate transformation on *se*(3), this allows us to transform spatial velocity from one coordinate to another. The Lie bracket is given by the matrix commutator ad_X_, which generalizes the standard cross-product operation in *se*(3).

(2) $${\mathrm{Ad}}_{g}\left(Y\right)=gYg^{-1}$$

(3) $${\mathrm{ad}}_{X}\left(Y\right)=\left[X,Y\right]=\mathrm{XY}-\mathrm{YX}$$

Writing **X** as column vector pair (*ω*, *v*), the 6 × 6 matrix representation of adjoint transformation and Lie bracket are

(4) $${\mathrm{Ad}}_{g}=\left[\begin{array}{cc} R & 0\\ \hat{p}R & R \end{array}\right],\;{\mathrm{ad}}_{X}=\left[\begin{array}{cc} \hat{\omega } & 0\\ \hat{v} & \hat{\omega } \end{array}\right]$$

Spatial moment-force pair $\left(m,f\right)\;m,f\in R^{3}$ are elements of the dual space of *se*(3), which is denoted as *se*(3)*. The *dual Adjoint map*${\mathrm{Ad}}_{g}^{*}:\mathrm{se}\left(3\right)*\to \mathrm{se}\left(3\right)*$ and Lie bracket ${\mathrm{ad}}_{X}^{*}$ are given by

(5) $${\mathrm{Ad}}_{g}^{*}=\left[\begin{array}{cc} R^{T} & -\hat{p}R^{T}\\ 0 & R^{T} \end{array}\right]\;{\mathrm{ad}}_{X}^{*}=\left[\begin{array}{cc} -\hat{\omega } & -\hat{v}\\ 0 & -\hat{\omega } \end{array}\right]$$

Let *V*_2_ = (*ω*_2_, *v*_2_) ∊ *se*(3) denote the spatial velocity of a rigid body in frame *M*_2_, g_12_ ∊ *SE*(3) represents the right coordinate transformation from frame *M*_1_ to frame *M*_2_, and then the spatial velocity relative to frame *M*_1_, denoted by *V*_1_ = (*ω*_1_, *v*_1_), is given by $V_{1}=Ad_{g_{12}}V_{2}$. Let *F*_2_ = (*m*_2_, *f*_2_) ∊ *se*(3) * denotes the moment-force pair acting on a rigid body with respect to frame *M*_2_, *g*_12_ ∊ *SE*(3) represents the right coordinate transformation from frame *M*_1_ to frame *M*_2_, and then the spatial force in frame *M*_1_ is given by $F_{2}=Ad_{g_{12}}^{*}F_{1}$.

On *SE*(3) and its proper subgroups, the exponential map *exp*: *se*(3) → *SE*(3) is a surjective map and a local diffeomorphism [21]. Given $\hat{\psi }\in \;\mathrm{so}\left(3\right)$ and $X=\left(\hat{\psi },q\right)\in \;\mathrm{se}\left(3\right)$, we have

(6) $$\exp_{\mathrm{SO}\left(3\right)}\left(\hat{\psi }\right)=I+\sin∥ \psi ∥ \frac{\hat{\psi }}{∥ \psi ∥ }+\left(1-\cos∥ \psi ∥ \right)\frac{{\hat{\psi }}^{2}}{{∥ \psi ∥ }^{2}}$$

(7) $$\exp_{\mathrm{SE}\left(3\right)}\left(X\right)=\left[\begin{array}{cc} \exp_{\mathrm{SO}\left(3\right)}\left(\hat{\psi }\right) & A\left(\psi \right)q\\ 0 & 1 \end{array}\right]$$

(8) $$A\left(\psi \right)=I+\left(\frac{1-\cos∥ \psi ∥ }{∥ \psi ∥ }\right)\frac{\hat{\psi }}{∥ \psi ∥ }+\left(1-\frac{\sin∥ \psi ∥ }{∥ \psi ∥ }\right)\frac{{\hat{\psi }}^{2}}{{∥ \psi ∥ }^{2}}$$

Equation (6) is also known as Rodrigues formula. The logarithmic map *log*: *SE*(3) → *se*(3) is the inverse operation of exponential map*.* Let (**R**, *p*) ∊ *SE*(3) be such that tr(R) ≠ −1, then

(9) $$\log_{\mathrm{SO}\left(3\right)}\left(R\right)=\frac{\phi }{\sin\phi }\left(R-R^{T}\right)\in \;\mathrm{so}\left(3\right)$$

in which *ϕ* satisfies $\phi =\frac{1}{2}\left(\mathrm{tr}\left(R\right)-1\right)$ and |*ϕ*| < *π*. Also

(10) $$\log_{SE\left(3\right)}\left(R,p\right)=\left[\begin{array}{cc} \hat{\psi } & A^{-1}\left(\psi \right)p\\ 0 & 0 \end{array}\right]$$

where $\hat{\psi }=\log_{\mathrm{SO}\left(3\right)}\left(R\right)$.

Note that element of the Lie algebra can be expressed as a velocity as in Eq.(1) or the logarithm coordinates in Eq. (10). We denote them with $V=\left(\hat{\omega },v\right)$ in the first case, and with $X=\left(\hat{\psi },q\right)$ in the second case. According Ball’s screws theory, every rigid coordinate transformation corresponds to a finite screw motion [22]. More precisely, the twist corresponds to the spatial velocity $V=\left(\hat{\omega },v\right)$ of the Lie algebra, a screw refers to the exponential coordinate $X=\left(\hat{\psi },q\right)$. The screw is an element of the 1–dimensional projective space of *se*(3), while a screw system with k degree of freedom (DOF) is a k–dimensional subspace [23]. The distance between the configuration state *g* and the identity e_G_ = **I**∈*SE*(3) is given by the norm of the logarithmic coordinate, which is an Ad-invariant metric on the matrix Lie group G by either left or right transformation [24].

(11) $${∥ g∥ }_{G}={\langle \log\left(g\right),\log\left(g\right)\rangle }^{1/2}=\frac{1}{2}\sqrt{\mathrm{tr}\left[\log{\left(g\right)}^{T}\log\left(g\right)\right]}$$

### Dynamic constrains and reference search

In deforming anatomic environment, the motion of instrument suffers from complex geometrical and dynamic constraints, their translational and rotational degree of freedom are restricted. MIS operation requires more sophisticated skills for the surgeons, making a distinction between and preferred and non-preferred directions and employed different control strategy helps reduce operation burden for surgeons. This can be achieved by construction of the holonomic/non-holonomic constraints and searching for the corresponded reference trajectories. The surgeons have complete control over the preferred directions, which are featured by a set of unit motion screws; while the deviation relative to the reference can be compensated autonomously. For a fully actuated control systems (the number of independent control inputs equals to the number of position variables), the reference trajectory falls into the holonomic and non-holonomic according to the constraints applied to the end-effector.

### (i). Holonomic constraint and the corresponded reference

In holonomic constraints, the DOF of reference equals to the dimension of *SE*(3), motion of the end-effector is strictly constrained. The task sequence is defined as a generalized time-varying curve *g*_*r*_(*λ*, *t*) ∊ *G*, *λ* ∊ *R*. Translation and rotation are continuous in *λ* − *t* plane, the first-order partial derivatives for *t* and *λ* exist. The temporal velocity with respect to inertia frame refers to

(12) $$V_{d}\left(\lambda ,t\right)=\frac{\partial g_{r}}{\partial t}g_{r}^{-1},\;V_{r}\in \;\mathrm{se}\left(3\right)$$

The tangent screw

(13) $$V_{t}\left(\lambda ,t\right)=\frac{\partial g_{r}}{\partial \lambda }g_{r}^{-1},\;V_{t}\in \;\mathrm{se}\left(3\right)$$

in inertia frame represents the preferred direction of instrument motion, which is independent of the choice of parameter *λ*. Given the current configuration *g* of the end-effector, the instantaneous desired configuration *g*_*d*_ on the task sequence *g*_*r*_(*λ*, *t*) refers to the one with minimum norm

(14) $$\begin{array}{l} e\left(\lambda \right)={\left(g_{r}{\left(\lambda ,t\right)}^{-1}g\left(t\right)\right|}_{t}\\ g_{d}=\arg\min_{\lambda }{∥ e\left(\lambda \right)∥ }_{G}=\arg\min_{\lambda }\frac{1}{2}\sqrt{2\psi^{T}\psi +q^{T}q} \end{array}$$

in which $\log_{\mathrm{SE}\left(3\right)}\left(e\right)=\left(\hat{\psi },q\right)\in \mathrm{se}\left(3\right)$, the norm of logarithmic error is a weighted distance of pure rotation and translation. Searching for the nearest reference along the task sequence can be generalized as the traversal searching the minimum point along the whole task sequence for initialization, local searching along the direction of norm reduction and the minimum is always reachable due to continuous restriction on rotation and translation.

### (ii). Non-holonomic constraint and the corresponded reference

In non-Holonomic constraints, the DOF of reference is less than the dimension of Lie group, such as pure translation *R*^3^, rotation *SO*(3), or other displacement subgroups, motion of the end-effector is partially constrained. For example, instrument motion on a deforming surface while the tool shaft along the norm direction, which refers to the displacements in *SE*(2). Given the reference *g*_*r*_ = ([*m*_*x*_, *m*_*y*_, *m*_*z*_], *p*_*r*_), *f*(*p*_*r*_) = 0 and current state *g* = ([*n*_*x*_, *n*_*y*_, *n*_*z*_], *p*), the task is to make *n*_*z*_ → *m*_*z*_, *p* → *p*_*r*_. Any rotation about axis *z* cannot change the direction of axis-z, the instantaneous reference *g*_*d*_ is the one with minimum norm in all possible frames *g*_*r*_*g*_z_(*θ*),

(15) $$\begin{array}{l} g_{z}\left(\theta \right)=(R_{z}(\theta ),0),\theta \in [0,\pi )\\ g_{r}\left(\theta \right)=g_{r}g_{z}(\theta )\\ e\left(\theta \right)=g_{r}(\theta ){}^{-1}g\\ g_{d}=\arg\min_{\theta }{∥ e\left(\theta \right)∥ }_{G}={\left(g_{r}\left(\theta \right)\right|}_{\mathrm{tr}\left(e\left(\theta \right)\right)=4} \end{array}$$

When the axis vectors *n*_*z*_ and *m*_*z*_ coincides, the rotation matrix of reference *g*_*d*_ equals to that of robot state *g* according to Eq.(15), that means the user has complete control over the robot along the tangent plane and directs the task flow intuitively, the deviations relative to reference can be compensated manually or autonomously during human-robot cooperation.

### Spatial compliance for virtual fixture on SE(3)

Admittance control facilitates VF by allowing the end-effectors to comply with the manual force anisotropically. The compliant behavior of admittance VF is featured by a 6 × 6 symmetric positive semidefinite (PSD) compliance matrix **C**_VF_ : *se*(3) * → *se*(3), which maps spatial force into small displacement [25,26]. The preferred and non-preferred directions construct the geometry constraints, both can be represented by unit screws in *se*(3). Given a set of linearly independent allowable screw set **S** in body-fixed frame, the task dependent compliant matrix $C_{\mathrm{VF}}^{b}$ can be decomposed into sum of *m* rank-1 PSD matrices,

(16) $$\begin{array}{l} S=\left\{S_{i}\right\},S_{i}={\left[\psi_{i}^{T},q_{i}^{T}\right]}^{T},i=1,\ldots m\leq 6\\ C_{\mathrm{VF}}^{b}=c_{1}S_{1}S_{1}^{T}+c_{2}S_{2}S_{2}^{T}+\cdot \cdot \cdot +c_{m}S_{m}S_{m}^{T},c_{i}>0\\ \;=C_{1}+C_{2}+\cdot \cdot \cdot +C_{m} \end{array}$$

positive scalar *c*_*i*_ defines the compliance for each primitive compliance matrix *C*_*i*_. A wrench applied about the screw yields a twist deformation along the same screw, high compliance is encountered along the preferred directions, while low compliance along the non-preferred directions. Making a distinction between and non-preferred directions helps reduce operation burden of surgeons, they only focus on operations along the preferred direction. Any deformation *δX*^*b*^ = (*δφ*^*b*^, *δq*^*b*^) complying with the manual force *F*_*h*_^*b*^ = (*m*^*b*^, *f*^*b*^) can be linearly represented by motion screws of **S**

(17) $$\left[\begin{array}{c} \delta \varphi^{b}\\ \delta q^{b} \end{array}\right]=C_{\mathrm{VF}}^{b}\left[\begin{array}{c} m^{b}\\ f^{b} \end{array}\right].$$

The term *F*_*h*_^*b*^ is manual force acting on the end-effector with respect to body frame, *δ***X**^*b*^ refers to the dual vectors of translational deformation and rotational deformation in body frame. A complete constraint corresponds to a full-rank compliance matrix **C**, a deficient-rank matrix indicates an incomplete constraint. The compliance matrix $C_{\mathrm{VF}}^{b}$ is defined in body-fixed frame ‘*b*’, if *g* ∈ *SE*(3) represents the right coordinate transformation from frame ‘*b*’ to frame ‘*a*’, then the compliance matrix $C_{\mathrm{VF}}^{a}$ in the new coordinate frame ‘*a*’ is given by

(18) $$C_{\mathrm{VF}}^{a}=Ad_{g^{‒1}}C_{\mathrm{VF}}^{b}Ad_{g^{‒1}}^{*}$$

A customized virtual fixture for complicated surgical tasks can be treated as the combination of one or more primitives [27]. In Table 1, we lists the preferred motion screws for four static primitive tasks. The featured screw system could be redundant, twist matrices intersection is a method to reduce the redundancy of multiple allowable screw sets [28,29].

**Table 1:** Four primitive tasks and the corresponding preferred motion screw

**Primitive tasks**	**Allowable motion screw set**
Fixed configuration	*g*_*d*_ ∊ *SE*(3), *S* = log (*g*_*d*_^−1^*g*)
Trajectory tracking *g*_*d*_ ∊ *SE*(3)	$$\begin{array}{l} S_{1}={\dot{g}}_{d}g_{d}^{-1}\\ S_{2}=\log\left(g_{d}^{-1}g\right) \end{array}$$
Rotate around a line	*S* = (*s*,*r* × *s*) ***s***: rotation axis, ***r***: arbitrary point on ***s***
Move on plane	*S*_1_ = (*0*,*v*_1_),*S*_2_ = (*0*,*v*_2_),*S*_3_ = (*s*,*r* × *s*) span{*v*_1_,*v*_2_}: continent plane, ***s***: norm direction

### Dynamic virtual fixture for admittance-type device

Considering that the end-effector is of admittance type, which can be modeled as non-backdrivable kinematic system, and all joints are equipped with velocity-source actuators. The VF on admittance type device acts as anisotropic compliant wall, the end-effector complies with the manual force along preferred the directions. Bettini and Marayong [5,7] combined the deviation error into the allowable motion subspace and redefined a new VF that makes it possible to reduce the deviations manually. However, it has been shown that manual compensation does not necessarily compensate for all deviations, especially when the VF is defined for translation and the deviation error is of orientation. Castillo-Cruces et al. [14] added linear error feedback term into the admittance controller to compensate rotation and translation deviations separately and autonomously along the non-preferred directions.

However, the first-order admittance controller with manual or autonomous error compensation is inadequate to adapt to dynamic environment. This section extends these VFs with dynamic tracking into a deforming environment on *SE*(3). The admittance-type robot can be modelled as a left invariant first-order fully actuated systems on *SE(3),* and satisfies the quasi-equilibrium condition ${\dot{V}}^{b}=0$.

(19) $$\begin{array}{l} \dot{g}=gV^{b}\\ V^{b}=\left(\omega^{b},v^{b}\right)\in \mathrm{se}(3)\\ \left[\begin{array}{c} \omega^{b}\\ v^{b} \end{array}\right]=K_{c}C_{\mathrm{VF}}\left(g,g_{d}\right)\left[\begin{array}{c} m^{b}\\ f^{b} \end{array}\right]+U_{\mathrm{DVF}}^{\mathrm{Ad}}(g,g_{d}) \end{array}$$

The VF is defined as task-dependent and time-varying compliance matrix $C_{\mathrm{VF}}\left(g,g_{d}\right)$, $C_{\mathrm{VF}}^{b}$ , velocity **V**^*b*^, and manual force $F^{b}=\left(m^{b},f^{b}\right)\in \mathrm{se}\left(3\right)*$ acting on the end-effector are represented in body-fixed frame. Admittance gain *K*_*c*_ =*diag*(*c*_*r*_*I*_3×3_, *c*_*p*_*I*_3×3_) controls the compliance of the robot to user input, choosing *c*_*r*_, *c*_*p*_ low imposes additional resistance along the preferred directions, high compliance could result in fast robot motion. Velocity of end-effector is controlled jointly by the admittance controller and dynamic tracking controller *U*_*DVF*_^*Ad*^ to make the robot state *g* track the reference *g*_*d*_, which is described by *ġ*_*d*_ = **V**_*d*_*g*_*d*_. The deviation of robot state *g* relative to the reference *g*_*d*_ with respect to the reference frame is defined as *e* = *g*_*d*_^−1^*g*, the matrix form is given by

(20) $$e=\left[\begin{array}{cc} R_{d}^{T}R & R_{d}^{T}\left(p-p_{d}\right)\\ 0 & 1 \end{array}\right]$$

It is also called natural error and independent of the choice of inertia frame [17]. Another equivalent definition is *e*^*b*^ = *g*^−1^*g*_*d*_, the deviation is seen in body frame. These two error definitions and their logarithmic coordinate are mutually invertible.

(21) $$\log\left(e\right)=-\log\left(e^{b}\right)$$

Define the configuration error *ė* = *e***V**_*e*_ as the first case, the error velocity with respect to inertia frame is given by

(22) $$V_{e}=-Ad_{g^{-1}}V_{d}+V^{b}$$

We design the dynamic tracking controller with logarithmic error feedback in matrix form

(23) $$U_{\mathrm{DVF}}^{\mathrm{Ad}}\left(g,g_{d}\right)=Ad_{g^{-1}}V_{d}-K_{p}\log\left(e\right)$$

where *K*_*p*_ = *k*_*p*_*D*_*e*_, *D*_*e*_ =*diag*(*k*_*r*_*I*_3×3_, *I*_3×3_), *k*_*p*_,*k*_*r*_ > 0, the linear coefficient *k*_*p*_ controls the speed of error convergence, maximum choice of *k*_*p*_ is restricted by the actuation power of motors, the rotation-translation (R-T) ratio *k*_*r*_ controls the synchronous the rotation and translation. We take this control law into the robot kinematics and cancel out manual effect, the error control function of the close loop system satisfies

(24) $$V_{e}+K_{p}\log\left(e\right)=0$$

The control law exponentially stabilizes the configuration error *e* at identity **I** from any initial configuration. Let *X* = log (*e*(*t*)) = (*ψ*, *q*) ∊ *se*(3) be the logarithmic coordinate of *e*(*t*), we can relate the column vector form *Ẋ* and *V*_*e*_ through

(25) $$\dot{X}=\sum_{n=0}^{\infty }\frac{{\left(-1\right)}^{n}B_{n}}{n!}ad_{X}^{n}\left(V_{e}\right)=B_{X}V_{e}$$

where the {B_n_} are Bernoulli numbers, the symbols B_x_ denote the Lie bracket series [17]. We have

B_X_ ***X*** = ***X*** and $B_{X}\left[\begin{array}{l} 0\\ q \end{array}\right]=\left[\begin{array}{l} 0\\ A{\left(\psi \right)}^{-T}q \end{array}\right]$

so

$$\dot{X}=-B_{X}\left(k_{p}k_{r}\left[\begin{array}{l} \psi \\ q \end{array}\right]+\left(k_{p}-k_{p}k_{r}\right)\left[\begin{array}{l} 0\\ q \end{array}\right]\right)=-k_{p}k_{r}X-\left(k_{p}-k_{p}k_{r}\right)\left[\begin{array}{l} 0\\ A{\left(\psi \right)}^{-T}q \end{array}\right]$$

Separating the rotational part and the translational parts

(26) $$\dot{\psi }=-k_{p}k_{r}\psi$$

(27) $$\begin{array}{l} \dot{q}=-k_{p}k_{r}q-k_{p}R_{\psi }q\\ R_{\psi }=\left(1-k_{r}\right)A{\left(\psi \right)}^{-T} \end{array}$$

the effect of rotation on the translation is featured by operator ℛ_***ψ***_, which is complex and highly dependent on the rotation axis *ψ*. We can adjust the interconnection between rotation and translation by choosing R-T ratio *k*_*r*_. The rotation part is exponentially stabilized for all initial rotation matrix **R**(0) such tr(**R**) ≠ −1. Regarding the translational part, consider the candidate Lyapunov function $W=\frac{1}{2}{∥ q∥ }^{2}$. Its time derivative satisfies

$$\begin{array}{l} \frac{d}{\mathrm{dt}}W=-\langle q,k_{p}k_{r}q+\left(k_{p}-k_{p}k_{r}\right)A{\left(\psi \right)}^{-T}q\rangle \\ \;=-k_{p}k_{r}{∥ q∥ }^{2}-\left(k_{p}-k_{p}k_{r}\right)\left({∥ q_{\left|\right|}∥ }^{2}+\alpha \left(∥ \psi ∥ \right){∥ q_{⟂}∥ }^{2}\right)\\ \;<0\; \end{array}$$

where *α*(*y*) ≜ (*y*/2) cot (*y*/2), 0 < ‖*ψ*‖ < 1, *q* = *q*_||_ + *q*_⟂_ is the orthogonal decomposition of ***q*** along span {*ψ*} and {*ψ*}_⟂_. Thus global exponential stability is proven also for translational part.

**Figure 1:**
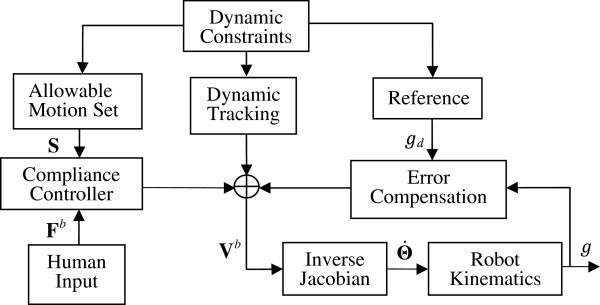
Control loop of DVF for admittance-type device.

Any deviation of the end-effector from the reference is compensated autonomously. This error compensation is regarded as a target tracing acting on the virtual fixture [14]. The discrete realization of the VF controller usually introduces time delay and dynamic tracking errors, forecast of the reference velocity **V**_*d*_ helps improve the tracking precision. As seen in Figure 1, the instantaneous reference and allowable motion set are defined from multiple geometric constraints; manual operation is regulated by the compliance controller, which is synthesized by the allowable motion set. The operator has complete control along the preferred directions, fast motion along the non-preferred direction and deviations relative to the reference are compensated autonomously. The dynamic tracking term in the DVF controller helps the surgeon to deal with operations in deforming environment.

## Experiments, results and discussion

The proposed DVF is designed for fully actuated robotic system with force/torque sensor. However, both force/torque sensor and admittance controlled device are not available. We designed a virtual proxy based hardware-in-the-loop simulation test bed to evaluate the proposed DVF. The PHANToM Omni device, from SensAble Technologies, is equipped with 3 degree-of-freedom (DOF) actuations and 6 DOF position measurements. It serves as a force source rather than a haptic device to drive a virtual admittance-type proxy alternatively. The device is actuated passively by human force that applied on the end-effector, while there is no haptic feedback to the operator.

The kinematics equation of the admittance-type proxy refers to

(28) $$\begin{array}{l} {\dot{g}}_{P}=g_{P}V_{P}^{b}\\ V_{P}^{b}=C_{\mathrm{VF}}\left(g,g_{d}\right)F^{b}+U_{\mathrm{DVF}}^{\mathrm{Ad}}(g,g_{d}) \end{array}$$

the proxy is controlled by the DVF controller in Eq. (19). The proxy complies with the actuation force *F*^*b*^ acting on the proxy with respect to body-fixed frame, which is estimated from the robot dynamics of the PHANToM device.

(29) $$\begin{array}{l} J^{T}\left(\Theta \right)F^{s}=M(\Theta )\ddot{\Theta }-N(\Theta ,\dot{\Theta })\dot{\Theta }\\ F^{b}=Ad_{g}^{*}F^{s} \end{array}$$

where **M**(***Θ***) is the inertia matrix, $N\left(\Theta ,\dot{\Theta }\right)$ is the Coriolis matrix, the gravity term **C**(***Θ***) is eliminated from the actual manual force, kinematics and dynamics details of the Phantom Omni device can be found in [30]. *F*^*s*^ represents the manual force with respect to the inertia frame, **J**(***Θ***) is manipulator Jacobian matrix, *U*_*DVF*_^*Ad*^ refers to the dynamic tracking controller in Eq. (23).

**Figure 2:**
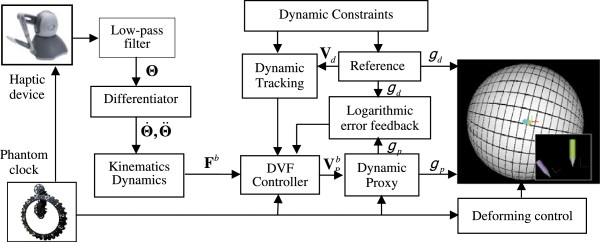
Hardware-in-the-loop test bed for DVF simulation on admittance-type proxy.

The real haptic device and the virtual admittance-type proxy are integrated in the MATLAB/Simulink environment by the PHANSIM TOOLKIT. The overview of the hardware-in-the-loop test bed for DVF implementation on admittance-type proxy is illustrated in Figure 2. The control loop of the haptic device runs at 1 kHz, we use the PHANToM clock to down sample the joint measurements to 200Hz. The joint angles **Θ** of the PHANToM Omni are processed by a low-pass filter and a differentiator successively to estimate the joint velocity $\dot{\Theta }$ and acceleration $\ddot{\Theta }$, manual force *F*^*b*^ are estimated by the Phantom robot dynamics in Eq. (29). The proxy state *g*_*P*_ is updated by proxy body velocity *V*_*P*_^*b*^ discretely, which is jointly controlled by admittance controller and dynamic error compensator Eq. (28). The proxy motion is constrained on a deforming sphere to simulate the beating heart surgery environment. We use an external clock to update the reference state *g*_*d*_, proxy state and the deforming radius. For fully apprehension, a 3D virtual environment is built up and visualized using the MATLAB Virtual Reality Toolbox. Both the proxy and the reference are modelled as rigid pens with unit isotropic inertia matrix.

In the simulation experiment, we simplify the admittance controller robot as a virtual proxy, manual force is estimated indirectly through robot dynamics rather than a real force sensor. The proxy workspace is not restricted compared to a real robot, measurement errors of joint angles are not involved, manual force is also inaccurate due to inertia parameters estimation. We will evaluate the proposed algorithm on a real admittance controlled device when all instruments are ready in the future.

Numerous surgical tasks require the surgeon to follow a predetermined path on a deformable anatomy tissue surface while maintaining the shaft orientation within a safe range, such as minimally invasive beating heart surgery, the area of interest deforms with heartbeat and respiratory motion, instrument motion should be regulated to satisfy the varying geometric constraints. We simplified this task as a non-holonomic constraint problem by moving a tool on a deforming ball, while the tool shaft along the radial direction, the axial rotation is not constrained. The radius r of the ball varies periodically, *r* = *r*_0_ + *Δr* sin *ωt*, *r*_0_ = 15 mm, *Δr* = 5 mm. The bandwidth of the position measurements is 26 Hz, which refers to the upper limit of tissue motion resulted from physiologic movements, such as heartbeat and respiration [16].

The instantaneous reference *g*_*d*_ refers to the one with minimum norm in Eq. (14). The allowable motions on the deforming sphere refer to translation along the tangent directions and rotation around the radius direction, the corresponded allowable motion set S is defined in the inertia frame,

$$S_{1}=\left(0,r_{x}\right),S_{2}=\left(0,r_{y}\right),S_{3}=\left(r_{z},p_{d}\times r_{z}\right),\;S=\left\{S_{1},S_{2},S_{3}\right\}$$

where *r*_*x*_, *r*_*y*_, *r*_*z*_ are unit axis vectors of *g*_*d*_, *p*_*d*_ is the translation part. The compliance matrix ***C***_VF_ is synthesized by the allowable motion screw set S. The workspace of the Phantom Omni device is 1:1 mapped into the proxy workspace, the corresponding coordinate axes are parallel and without rotational transformation. The initial state of the proxy tool is set to be the inkwell-calibrated configuration. The translation and orientation errors of the tool with respect to the reference are defined as

(30) $$\begin{array}{l} e=g_{d}^{-1}g=\left(R_{d}^{T}R,R_{d}^{T}\left(p-p_{d}\right)\right)\\ e_{p}=∥ p-p_{d}∥ \\ e_{r}=∥ \psi ∥ \; \end{array}$$

Rotational error *e*_*r*_ describes the angle of the rotation about the equivalent axis **ψ**, translational error *e*_*p*_ refers to the distance between tool tip ***p*** and reference *p*_*d*_. We use tracking error and following index to evaluate the performance of the proposed dynamic DVF in deforming environment. Translational tracking error (TTE) and rotational tracking error (RTE) refer to the root-mean-square of translation and orientation errors respectively during the human-robot cooperation. The following index represents how quick and how well the proxy can follow manual operation, or how much manual input can be transferred to the task related commands. More specifically, the following index is defined as the cross-correlation coefficient of manual force *F*^*b*^ = (*m*^*b*^, *f*^*b*^) and corresponded velocity acted on the proxy *V*_*C*_^*b*^ = (*ω*_*C*_^*b*^, *v*_*C*_^*b*^), which consists of the admittance controller output and the logarithmic error feedback in Eq. (22). The rotational following index (RFI) and translational following index (TFI) for admittance-type device are defined as

(31) $$\mathrm{RFI}=\mathrm{corr}\left(\omega_{C}^{b},m^{b}\right)=\frac{\sum {\left(\omega_{C}^{b}-{\bar{\omega }}_{C}^{b}\right)}^{T}\left(m^{b}-{\bar{m}}^{b}\right)}{\sqrt{\sum {∥ \omega_{C}^{b}-{\bar{\omega }}_{C}^{b}∥ }^{2}\sum {∥ m^{b}-{\bar{m}}^{b}∥ }^{2}}}$$

(32) $$\mathrm{TFI}=\mathrm{corr}\left(v_{C}^{b},f^{b}\right)=\frac{\sum {\left(v_{C}^{b}-{\bar{v}}_{C}^{b}\right)}^{T}\left(f^{b}-{\bar{f}}^{b}\right)}{\sqrt{\sum {∥ v_{C}^{b}-{\bar{v}}_{C}^{b}∥ }^{2}\sum {∥ f^{b}-{\bar{f}}^{b}∥ }^{2}}}$$

Three experiments were conducted to validate the efficiency of proposed DVF for admittance-type proxy.

### Proportional feedback control experiment

In this experiment, we compare the performance of three control laws to stabilizes the robot state *g* at fixed state *g*_*d*_ from any initial state *g*(0) = (*R*(0), *p*(0)). The deviation of the end-effector state relative to the reference is defined as *e* = *g*_*d*_^−1^*g*, the logarithmic coordinates refers to $\log\left(e\right)=\left(\psi ,q\right)\in \mathrm{se}\left(3\right)$.

1). Separate feedback

$$\left[\begin{array}{c} \omega^{b}\\ v^{b} \end{array}\right]=-\left[\begin{array}{cc} k_{w}I_{3\times 3} & 0\\ 0 & k_{v}I_{3\times 3} \end{array}\right]\left[\begin{array}{l} \psi \\ p-p_{d} \end{array}\right]$$

2). Double-geodesic feedback

$$\left[\begin{array}{c} \omega^{b}\\ v^{b} \end{array}\right]=-\left[\begin{array}{cc} k_{w}I_{3\times 3} & 0\\ 0 & k_{v}I_{3\times 3} \end{array}\right]\left[\begin{array}{l} \psi \\ R^{T}\left(p-p_{d}\right) \end{array}\right]$$

3). Logarithmic feedback

$$\left[\begin{array}{c} \omega^{b}\\ v^{b} \end{array}\right]=-\left[\begin{array}{cc} k_{w}I_{3\times 3} & 0\\ 0 & k_{v}I_{3\times 3} \end{array}\right]\left[\begin{array}{l} \psi \\ q \end{array}\right]$$

Law-1 takes the error definition and feedback control from Castillo-Cruces’s work [14], which treats translation and rotation separately. Law-2 applies proportional feedback actions along geodesic directions for both rotation and translation in *SO*(3) and *R*^3^ respectively, law-3 is the logarithmic feedback control. Law-1 is dependent on the choice of inertia frame, both law-2 and law-3 are independent of inertia frame choice. To compare the proportional feedback laws presented above, the same planar motion *SE*(2), initial state *g*_0_, destination state *g*_*d*_ and proportional parameters *k*_*w*_, *k*_*v*_ are set for the three control laws in our experiment.

**Figure 3:**
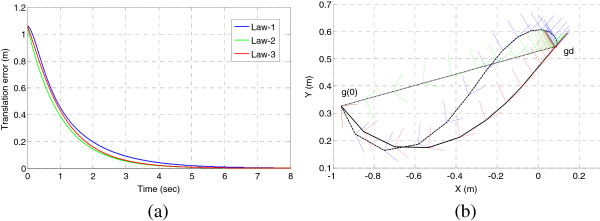
**Three proportional actions on SE(2) for 1st kinematics system with *k***_***w***_ **=** **2, *k***_***v***_ **=** **1, (a) translation error, (b) convergence trajectories, each point is depicted as a frame.**

As seen in Table 2, there are not obvious difference in the residual errors of the three laws, expect for their behaviors during the convergence period. All three control laws converge at the same rate in rotational part, as seen in Figure 3, law-1 shows more curved behaviour and takes the longest path to approach the fixed state, law-2 follows the shortest path. The performance of Law-2 deteriorates for 2^nd^ order robotic systems, only the logarithmic feedback control could locally exponentially stabilized the robot state at **I**, more detail comparison can be found in [17]. We take the logarithmic feedback law in our DVF controller design, with which the robot performs steadily stable, both the rotational and translational part converged uniformly to zero, no oscillations appeared.

**Table 2:** Residual errors of three proportional feedback control laws

	**Law-1**	**Law-2**	**Law-3**
TTE (mm)	0.0046	0.0050	0.012
RTE (rad)	2.7108e-020	1.7837e-017	1.2123e-007

**Figure 4:**
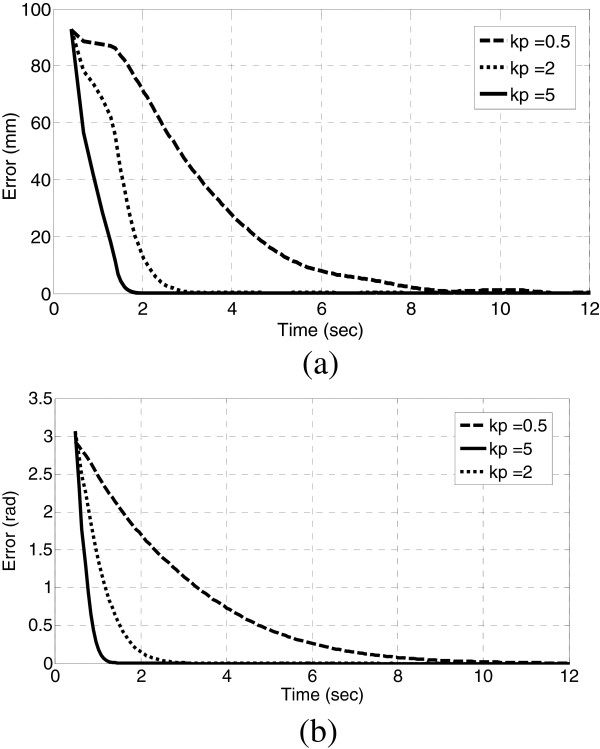
**Influence of *k***_***p ***_**on error convergence with deforming frequency 2 rad/s, R/T ratio =1, (a) translational error, (b) rotational error.**

We also analyzed the influence of proportional parameter *k*_*p*_ of the logarithmic error feedback controller. The proxy is asked to approximate the nearest reference on the sphere autonomously and manual excitation is still. The initial state of the proxy is set to be the inkwell-calibrated position. As shown in Figure 4, *k*_*p*_ controls the speed of error convergence, maximum choice of *k*_*p*_ is restricted by the actuation power of motors

### Dynamic tracking experiment

**Figure 5:**
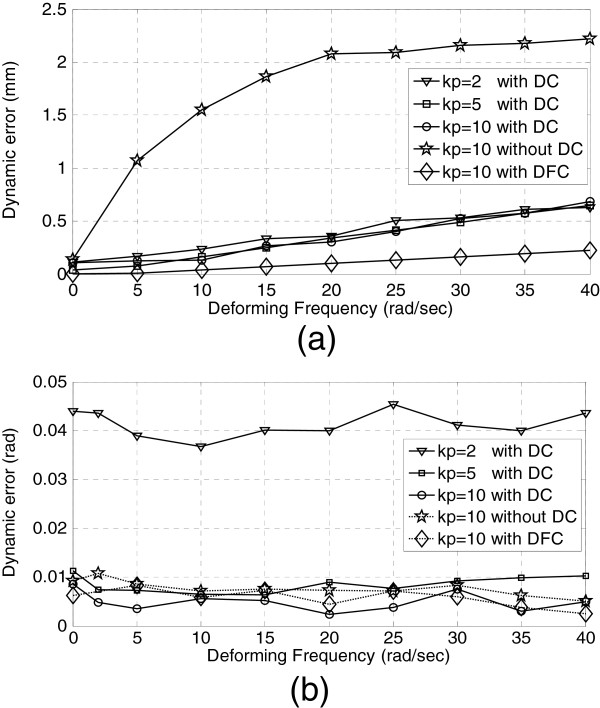
**Results of dynamic tracking experiment with difference *k***_***p ***_**settings and different tracking strategies (without dynamic compensation (DC), with dynamic compensation, with dynamic forecasting compensation (DFC)), (a) translational tracking error; (b) rotational tracking error.**

To discusses the dynamic properties, the operator dragged the gimbal pen of haptic device back and forth along x-axis in the inertia frame, the tangent components of manual force drove the proxy move along the circle of latitude on the deforming sphere with frequency varies from 0 to 40 rad/s, R-T ratio =1, *c*_*r*_ =0.1 rad/s, *c*_*p*_ =100 mm/s. As shown in Figure 5, the TTE increases linearly with deforming frequency growth, the impact of *k*_*p*_ on the dynamic error is not clear, but the impact of dynamic compensation (DC) is obvious. The RTE is suppressed by linear logarithm error compensation, and is independent of deforming frequency. This is due to the fact that orientation of reference remained constant while translation along the radial direction, translational deviation along the same direction is compensated autonomously; the proxy is actuated manually along tangent directions, the referred orientation varied accordingly. The discrete realization of the DVF controller usually introduces time delay and dynamic tracking error, forecast of the reference velocity **V**_*d*_ helps improve the tracking precision.

As seen in Figure 5, the TTE of the VF with DC is smaller than the VF without DC; furthermore, the VF with dynamic forecasting compensation (DFT) performs much better than the VF with DT. The DVF can improve the dynamic properties in low-frequency range, its dynamic performance deteriorated slowly with deforming frequency growth, this is due to the first-order kinematics property of the admittance-type device. R-T ratio can affect the synchronous of rotation and translation as well as the tracking error convergence speed because of interconnection between rotation and translation on *SE*(3). We recorded the TTE and settling time with deforming frequency 40 rad/s, *k*_*p*_ =20, admittance gains *c*_*r*_ =0.1 rad/s, *c*_*p*_ =100 mm/s, R-T ratio was set as 4,3,2,1,0.5 respectively, the results are listed in Table 3. Appropriate settings of R-T ratio help speed up the error convergence and reduce error level.

**Table 3:** Impact of R/T ratio on dynamic tracking error

**R-T ratio**	**4**	**3**	**2**	**1**	**0.5**
Tracking error(mm)	0.5347	0.5996	0.6487	0.6610	0.6569
Settling time(sec)	0.1	0.2	0.35	0.6	1.3

### Human-robot collaboration experiment

**Figure 6:**
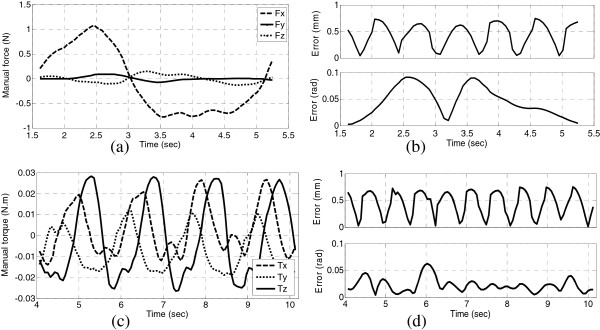
**The tracking error of multiple manual operations with *k***_***p***_ **=** **2, *ω*** **= 5 rad/s, R-T ratio = 1, *c***_***r***_ **= 0.1 rad/s, *c***_***p***_ **= 100 mm/s; (a) manual force when moving along axis-x; (b) resulted error of operation (a), (c) manual torque when pivoting around arbitrary axis; (d) resulted error of operation (c).**

This experiment is designed to analyze human factors on human-robot collaboration with assistance of the DVF controller. To analyze the influence of manual force on the tracking error, three people took part in this experiment. The operator applied time-varied force along x-axis with respect to inertia frame, manual force and tracking error are depicted in Figure 6(a-b); the operator pivoted the gimbal pen around arbitrary axis, the tool shaft orientation deviated from the reference accordingly, manual torque and the tracking error are plotted in Figure (c-d).

As seen in Figure 6, changes in the magnitude and direction of manual force had influence on rotational and translational errors. The operator dragged the tool along 8-shaped curve with three intensities of manual force{low, medium, high}, *k*_*p*_ =10, deforming frequency 5 rad/s, the following index RFI and TFI, dynamic tracking error TTE and RTE are recorded in Table 3.

As seen in Table 3, the dynamic tracking error is linearly related to manual force intensity and R-T ratio, the compliances *c*_*r*_, *c*_*p*_ play the same role as they scaled the manual force intensity that acted on the proxy. Faster manual operation resulted in larger dynamic tracking error. The proportional gain of the rotational part is multiplied by R-T ratio. The VF controller dominates the rotation over manual operation when large R-T ratio is set, the angular velocity resulted from human input is less than that from the logarithmic error feedback controller, and then the rotational following index decreases accordingly. A fair distribution on rotation control between the autonomous controller and human operation is achieved when the R-T ratio is low; the rotational following index has little change. The translational following index is negatively related to the R-T ratio, this is because the impact of rotation part on the translation is weakened when small R-T ratio is set. As seen from Tables 3 and 4, we can also conclude that higher R-T ratio helps suppress the dynamic tracking error, however it could decrease the rotational following index during human-robot cooperation, choosing R-T ratio small can increase the tracking error and the translational following index, the choice of R-T ratio should compromise between user experience and the tracking error. More specifically, the inter-relationship between the components of manual moment-force pair (**m**^*b*^, **f**^*b*^) and the output velocity (*ω*_*C*_^*b*^, *v*_*C*_^*b*^) are featured by the correlation coefficients of *F*^*b*^ and **V**_*C*_^*b*^. The operator is asked to drag the tool randomly on the deforming sphere for five minutes with deforming frequency 5 rad/s, *k*_*p*_ =10. All the data are standardized to eliminate the variable dimension difference. The Pearson product–moment correlation coefficients are calculated for all one-to-one element combinations in *F*^*b*^ and **V**_*C*_^*b*^ with confidence interval 99% [31].

**Table 4:** Results of shape-8 operation with assistance of DVF on admittance proxy

**Intensity**	**R-T ratio**	**RFI**	**TFI**	**RTE (rad)**	**TTE (mm)**
Low	4	0.1296	0.6148	0.0093	0.1046
Low	2	0.8138	0.8333	0.0260	0.1693
Low	1	0.6554	0.8647	0.0575	0.2633
Low	0.5	0.8008	0.9087	0.0856	0.3404
Medium	4	0.1815	0.7453	0.0180	0.1567
Medium	2	0.7633	0.9374	0.0452	0.1876
Medium	1	0.6231	0.8815	0.0712	0.2847
Medium	0.5	0.7000	0.9407	0.1226	0.3455
High	4	0.1692	0.8519	0.0291	0.1140
High	2	0.8190	0.9440	0.0727	0.1832
High	1	0.7173	0.7725	0.0812	0.2728
High	0.5	0.6133	0.8714	0.1755	0.3303

**Figure 7:**
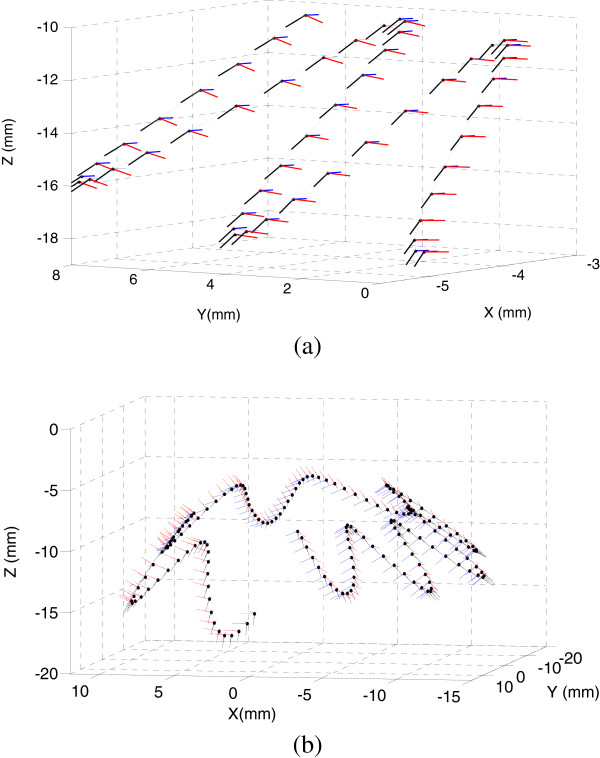
**Trajectories of the proxy along straight line (a) and circle (b) on the deforming sphere with *k***_***p***_ **= 5, *ω*** **= 5 rad/s, R-T ratio = 1, *c***_***r***_ **= 0.1 rad/s, *c***_***p***_ **= 100 mm/s.**

As seen in Table 5, the difference of correlation coefficient reveals that the DVF controller has stronger control over the manual operation in tool shaft orientation. Rotation ω_z_  round the tool shaft and translation v_x_, v_y_  along tangent directions are strictly linear correlative to manual input m_z_, f_x_, f_y_  respectively, the operator has completely control over these direction. The correlation between manual moment m_x_, m_y_  and tangent velocity v_x_, v_y_ are notable due to the coupling between rotation and translation. Velocity v_z_ is independent of the manual input; the DVF controller takes charge of the deviation compensation along the deforming direction.The trajectories of the operator driving the proxy along straight line and circle on the deforming sphere are illustrated in Figure 7, the complicated behaviors of dynamic task is beyond human control, making a distinction between the preferred and non-preferred directions and employed different control strategy helps reduce operation burden of surgeons and improve their skills in dynamic tasks.

**Table 5:** The correlation between manual force and proxy velocity

**Corr.**	**m**_**x**_	**m**_**y**_	**m**_**z**_	**f**_**x**_	**f**_**y**_	**f**_**z**_
ω_x_	0. 761	−0.192	−0.051	−0.003	−0.940	−0.692
ω_y_	−0.046	0.880	0.092	0.922	−0.058	0.114
ω_z_	−0.545	−0.043	1.000	0.173	0.048	0.021
v_x_	−0.127	0.889	0.134	0.998	−0.040	0.106
v_y_	−0.754	0.127	0.091	−0.044	0.998	0.676
v_z_	0.006	0.039	0.016	0.079	−0.029	0.012

Pearson's correlation coefficient with confidence interval 99%.

## Conclusions

This paper described a novel framework of DVF for admittance-type manipulators on the Euclidean Group *SE(3)* to assist the surgeons to deal with the dynamic tasks, which unites rotation and translation in a compact form We constructed the holonomic/ non-holonomic constraints, and then searched for the corresponded references to make a distinction between preferred and non-preferred directions. Different control strategies are employed to deal with the task along these directions. The DVF can improve the dynamic properties of human-robot cooperation in low-frequency deforming environment, and maintain synergy of orientation and translation during the operation. The experiments show that the DVF implemented on the virtual admittance-type proxy can assist the user to deal with the skilled operations in deforming environment. We will evaluate the proposed algorithm on a real admittance controlled device when all instruments are ready in the future.

## Notation and nomenclature

*a*; scalar

***f***; force vector

***m***; moment vector

***ω***; angular velocity vector

***v***; translational velocity vector

**R**; rotation matrix

***0***; null vector

**0**; null matrix

***q***; Vector

$\hat{q}$; cross-product matrix of vector ***q***

*SE*(3); Lie group

*SE*(2); Displacement subgroup of Lie group

*SO*(3); Rotation group

*se* (3); Lie algebra

*so*(3); Lie algebra of rotation group

*se*(3)*; dual space of Lie algebra

*so*(3)*; dual space of *so*(3)

*g*; elements in *SE*(3)

$V=\left(\hat{\omega },v\right)$; matrix form velocity in se(3)

*V* = (*ω*, *v*); column vector form velocity in se(3)

$X=\left(\hat{\psi },q\right)$; the matrix form logarithmic coordinates of *g*

*X* = (*ψ*, *q*); column vector form logarithmic coordinates of *g*

* ^*b*^; element relative to the body frame

* ^s^; element relative to the spatial frame

${\mathrm{Ad}}_{g},\;{\mathrm{Ad}}_{g}^{*}$; adjoint transformation

${\mathrm{ad}}_{X},\;{\mathrm{ad}}_{X}^{*}$; Lie bracket

exp _*SE*(3)_( * ); exponential map on *SE*(3)

log_*S*E(3)_(∗); logarithmic map on *SE*(3)

‖ ∗ ‖_G_; norm on the matrix Lie Group *SE*(3)

Generally, we use italic small characters for scalars, italic bold for vectors , bold capitals for matrices, italic bold capitals for vectors of Lie algebra.

## Competing interests

The authors declare that they have no competing interests.

## Authors’ contributions

DWZ proposed the framework of dynamic virtual fixture on *SE*(3) for admittance-type device and designed the hardware-in-the-loop experiment, JX derived the dynamics of PHANToM Omni for manual force estimation, QSZ participated in simulation experiments, LW revised the manuscript and contributed to the result discussion. All authors checked and approved the final manuscript.
